# Anabolic Heterogeneity Following Resistance Training: A Role for Circadian Rhythm?

**DOI:** 10.3389/fphys.2018.00569

**Published:** 2018-05-23

**Authors:** Donny M. Camera

**Affiliations:** Exercise and Nutrition Research Program, Mary MacKillop Institute for Health Research, Australian Catholic University, Melbourne, VIC, Australia

**Keywords:** molecular clock, muscle growth, responder, skeletal muscle, exercise adaptation

## Abstract

It is now well established that resistance exercise stimulates muscle protein synthesis and promotes gains in muscle mass and strength. However, considerable variability exists following standardized resistance training programs in the magnitude of muscle cross-sectional area and strength responses from one individual to another. Several studies have recently posited that alterations in satellite cell population, myogenic gene expression and microRNAs may contribute to individual variability in anabolic adaptation. One emerging factor that may also explain the variability in responses to resistance exercise is circadian rhythms and underlying molecular clock signals. The molecular clock is found in most cells within the body, including skeletal muscle, and principally functions to optimize the timing of specific cellular events around a 24 h cycle. Accumulating evidence investigating the skeletal muscle molecular clock indicates that exercise-induced contraction and its timing may regulate gene expression and protein synthesis responses which, over time, can influence and modulate key physiological responses such as muscle hypertrophy and increased strength. Therefore, the circadian clock may play a key role in the heterogeneous anabolic responses with resistance exercise. The central aim of this Hypothesis and Theory is to discuss and propose the potential interplay between the circadian molecular clock and established molecular mechanisms mediating muscle anabolic responses with resistance training. This article begins with a current review of the mechanisms associated with the heterogeneity in muscle anabolism with resistance training before introducing the molecular pathways regulating circadian function in skeletal muscle. Recent work showing members of the core molecular clock system can regulate myogenic and translational signaling pathways is also discussed, forming the basis for a possible role of the circadian clock in the variable anabolic responses with resistance exercise.

## Introduction

Skeletal muscle encompasses ~40% of total bodily mass and is a highly malleable tissue with the capacity to alter its structure and metabolism in response to internal and external stress signals such as muscle contraction and nutrition (Hawley et al., 2014; Camera et al., 2016). Moreover, skeletal muscle has been established to be an endocrine tissue through the section of various myokines that function both locally on the muscle tissue and on other tissues (Pedersen and Febbraio, 2012). As muscle proteins are continually degraded and damaged, turnover through muscle protein synthesis and breakdown processes are necessary to maintain protein quality and function. Maintaining the balance between these two processes is regulated by levels of physical activity and nutrition (Rasmussen and Phillips, 2003).

Physical activity, which includes any bodily movement produced by skeletal muscles, forms an integral part of human life that influences overall health across the lifespan (Hawley et al., 2014). In particular, the positive effects of load-bearing resistance exercise on human health are profound and unequivocal as evidenced by increased muscle mass, improved cognitive performance, enhanced insulin sensitivity and pre-servation of musculoskeletal function during aging (Hawley et al., 2014; Camera et al., 2016). These health benefits are crucial to the delay in sarcopenia which is the continual decline in muscle mass and functional capacity with age. Indeed, maintaining skeletal muscle function and health over a lifespan underpins daily physical work capacity (Rasmussen and Phillips, 2003). However, despite these proven health benefits, considerable variability in the magnitude of response in type I and type II muscle fiber cross-sectional area (Bamman et al., 2007), muscle cross-sectional area (Hubal et al., 2005; Hartman et al., 2007; Raue et al., 2012), lean body mass (Davidsen et al., 2011), one-repetition maximum strength (Hubal et al., 2005; Hartman et al., 2007; Phillips et al., 2013), and isometric strength (Hubal et al., 2005) exists from one individual to another to standardized resistance training programs. Perhaps the most established example of this variation in adaptation responses with resistance exercise is in the landmark Functional Polymorphisms Associated with Human Muscle Size and Strength (FAMuSS) study, where changes in muscle size ranged from 3% up to almost 60%, a 20-fold difference, following 12 weeks resistance training in healthy young adults (Hubal et al., 2005).

Heterogeneity or variability in exercise adaptation responses (whether it be resistance or endurance training) has led to the development of terms such as “non,” “low,” “moderate,” “high,” and “extreme” responders to describe the divergent magnitude in response of a particular individual to a given exercise stimulus (Camera et al., 2016). However, such terminology is subjective and depending on the statistical procedures (if any) used to classify/ segregate participants, can have no physiological or mechanistic basis. As Booth and Laye correctly communicate, the generalized term “non-responder” can be misleading as “it implies that no exercise-induced adaptations occur” (Booth and Laye, 2010). This point was clearly supported in a retrospective analysis study by the van Loon group that demonstrated participants were able to positively respond to at least one training outcome (i.e., lean body mass, muscle fiber size, strength, or physical work function) following 24 weeks resistance training in older (>65 years) men and women (Churchward-Venne et al., 2015). Nonetheless, these findings do not explain the basis for why variability still existed within each of these specific adaptation responses. For instance, if resistance training is well purported to increase muscle strength (Rhea et al., 2003; Peterson et al., 2010), why then was there still an 88% (+3 to +91%) and 152% (−39 to 113%) variability in leg extension strength responses in males and females, respectively, over the course of the 24-week training program from this retrospective analysis? The common counter-argument to this question usually centers on the specifics of the resistance exercise stimulus employed (number of sets, repetitions, volume, exercises performed, etc.) not being congruent to every individual (i.e., not a “one size fits all approach”) and that to achieve significant anabolic adaptation such individuals may require an alternative exercise prescription, extended training duration or volume (Montero and Lundby, 2017). It is also plausible, perhaps likely, that the “one-off” nature of strength and performance testing may explain some variability, particularly when considering the capacity for fatigue, tiredness and illness (etc.) to influence such measures (Sarzynski et al., 2017). Still, in light of an older, untrained cohort undertaking a lengthy and supervised resistance training program in this retrospective study, it could still be assumed that the “ceiling” for improvement in these individuals would be high to induce significant improvements greater than the minimum percentage changes observed.

## Mechanistic basis for heterogeneity in anabolic-related adaptation responses with resistance training

Compared to the multitude of studies investigating heterogeneity in VO_2max_ and aerobic capacity responses with endurance training (Lortie et al., 1984; Bouchard et al., 1999, 2011; Timmons et al., 2010), considerably less work has focused on the molecular basis for diversity in muscle strength and hypertrophy responses following resistance training. Nonetheless, several studies have provided insight to the potential molecular mechanisms that may underpin variability in anabolic adaptation responses previously observed with resistance training (Camera et al., 2016; Bamman et al., 2017). One of the first genetic markers implicated in the anabolic variability in muscle strength/hypertrophy with resistance training was α-actinin 3 (ACTN3). ACTN3, which belongs to a family of actin-binding proteins integral to binding and anchoring actin filaments, has been linked on multiple occasions to enhanced VO_2peak_ performance, with the frequency of the ACTN3 577X allele previously demonstrated to be overrepresented in endurance athletes (Yang et al., 2003). Using samples from the FAMuSS study, Clarkson and colleagues showed untrained women (aged 18–40 years) homozygous for the ACTN3 577X allele exhibited lower baseline maximal voluntary contraction compared with women who possessed the heterozygote genotype (Yang et al., 2003). In contrast, women homozygous for the mutant allele (577X) demonstrated greater absolute and relative 1-repetition maximum gains compared with the homozygous wild type (RR) after a 12-week forearm flexor and extensor resistance training program (also when adjusted for body mass and age). In men, no association between ACTN3 R577X genotype and muscle phenotype was observed (Yang et al., 2003). While these findings indicate a potential gender specific role for the ACTN3 577X allele in the anabolic responsiveness to resistance training, there are several limitations with the training intervention that may also contribute, in part, to the variability in responses observed. For instance, the resistance exercise program was only limited to elbow flexor/extensor resistance training exercises of the non-dominant arm and there was no apparent nutritional support to enhance anabolic adaptation responses.

The Bamman group has published several insightful papers investigating different aspects of the molecular mechanisms implicated in heterogeneous anabolic responses with resistance training. These studies have incorporated statistical (K-means) cluster analysis as a method to group participants into “extreme,” “modest,” and “non-responder” clusters based on changes in *vastus lateralis* mean myofiber cross-sectional area to a chronic resistance training program. The K-means cluster analysis approach is used to identify potential mechanisms underlying human intervention responsiveness in an unbiased manner (Bamman et al., 2007). Bamman et al. first used this approach when they reported large interindividual variability in myofiber hypertrophy following a 16-week resistance training program in untrained, but otherwise healthy, older (60–75 years), and younger (20–35 years) adults (Bamman et al., 2007). The observed heterogeneity was associated with altered myogenic gene expression (mechanogrowth factor and myogenin) across the three different clusters (i.e., “extreme,” “modest,” and “non-responder”) suggesting the change in magnitude of myofiber cross-sectional area is influenced by the increased expression of these genes. Using the same group of participants for analysis, it was later observed the number of satellite cells (muscle stem cells) increased only in “extreme” responders (Petrella et al., 2008). The increase in satellite cell number was concomitant with more myonuclei per fiber and an expanded myonuclear domain than “non-responders,” indicating the necessity for myonuclear addition through satellite cell activation to achieve substantial myofiber hypertrophy with resistance training. Another pivotal finding from this study was that basal (i.e., pre-training) satellite cell population was greater in the extreme cluster compared to the moderate and non-responder cohorts, suggesting extreme responders exhibit superior myogenic potential. This finding was subsequently supported with genomic microarray analysis which identified over 8,000 differentially expressed gene transcripts associated with transcriptional regulation and skeletal muscle development between “extreme” and “non-responder” clusters (Thalacker-Mercer et al., 2013). Of note, two key myogenic regulatory factors, Myogenic Differentiation (MyoD) and myogenin, displayed higher expression in the “extreme” cluster participants (Thalacker-Mercer et al., 2013).

The same research group recently reported that altered ribosome biogenesis responses may be implicated in regulating the extent of myofiber hypertrophy with resistance training (Stec et al., 2016). In a group of 42 older adults who performed a 4 week lower body resistance training program, K-means cluster analysis was again used to segregate participants into “extreme,” “moderate,” and “non-responders” based on changes in type II myofiber hypertrophy. In addition to the 83% increase in type II fiber cross sectional area observed post-training, “extreme” responders also significantly increased ribosomal RNA content suggesting ribosome biogenesis facilitates extreme hypertrophy in these individuals (Stec et al., 2016). The expression profile of specific microRNAs (miRNAs) may also be implicated in the variation in muscle growth responses with resistance training (Davidsen et al., 2011; Ogasawara et al., 2016). MicroRNAs are small (~20–30 nucleotides) non-coding ribonucleic acids (RNAs) that can promote mRNA (mRNA) degradation or suppress protein translation (He and Hannon, 2004). Thus, miRNAs possess the capacity to mediate changes in expression levels of particular mRNAs targets central to anabolic-related adaptations with resistance training. Recent work has showed altered miRNA expression profiles between “high” and “low” responders at baseline, as well as following acute and chronic resistance training programs in human skeletal muscle (Davidsen et al., 2011; Ogasawara et al., 2016). Thus, it would appear that the expression of particular miRNAs are involved in the magnitude of skeletal muscle anabolic adaptations with resistance training although much more work related to target validation of these miRNAs is still required. Another emerging factor with limited investigation that may be implicated in the heterogeneity in anabolic responses with resistance training is the influence of circadian rhythm. Recent work is now indicating that circadian rhythm, and the underlying circadian (or molecular) clock, is a central temporal regulatory mechanism involved in modulating skeletal muscle function and possible molecular and physiological responses to exercise training (Schroder and Esser, 2013; Chatterjee and Ma, 2016). This aspect of exercise and muscle physiology is largely unconsidered within the research field despite the potential for the circadian clock to modulate adaptation responses with resistance training.

## Circadian rhythms and regulation of the molecular clock

Multiple physiological functions (i.e., sleep wake cycle, body temperature, hormone secretion, and locomotor activity) as well as intracellular processes (mitochondrial metabolism, protein expression, enzyme activity, cell regeneration) are temporally coordinated into rhythms coinciding with the 24-h solar cycle (Bass and Takahashi, 2010; Peek et al., 2013). These circadian rhythms are present in most organisms ranging from single cell bacteria to plants, animals and humans. Such rhythms principally function to support “predictive homeostasis” by increasing metabolic capacity and select self-defense mechanisms in anticipation of periods of increased demand (Idda et al., 2012; Loudon, 2012). The ability to synchronize an endogenous rhythm with an environmental time cue provides a biological advantage when performing daily activities (Feillet et al., 2006).

Circadian rhythms function independently of external environmental stimuli however can be “entrained” by light exposure as well as other external cues such as scheduled feeding and exercise such that they are reset or modulated to be aligned with the timing of the specific environmental cue (Roenneberg et al., 2003). In humans, a central pacemaker located in the suprachiasmatic nucleus (SCN) of the hypothalamus co-ordinates the circadian clocks in multiple tissues through neural and hormonal pathways (Golombek and Rosenstein, 2010). The central clock in the SCN controls the secretion of the chief circadian hormone, melatonin, which acts principally on cell surface receptors within the central nervous system to regulate the sleep/wake cycle, as well as on peripheral tissues such as the liver and skeletal muscle, to stabilize their circadian rhythms (Golombek and Rosenstein, 2010). Light is the major entraining factor for the SCN while peripheral clocks are regulated indirectly by photic stimulation from the central clock in the SCN using neuro-humoral and temperature signals (Brown et al., 2002; Abraham et al., 2010) as well as a number of external cues, called Zeitgebers (German for time givers) such as scheduled feeding, exercise, temperature, social interactions and pharmacological manipulation, in an SCN-independent manner (Damiola et al., 2000; Tahara and Shibata, 2013). Thus, peripheral tissues also contain their own molecular clock mechanism that regulate local physiological functions.

**Figure 1:**
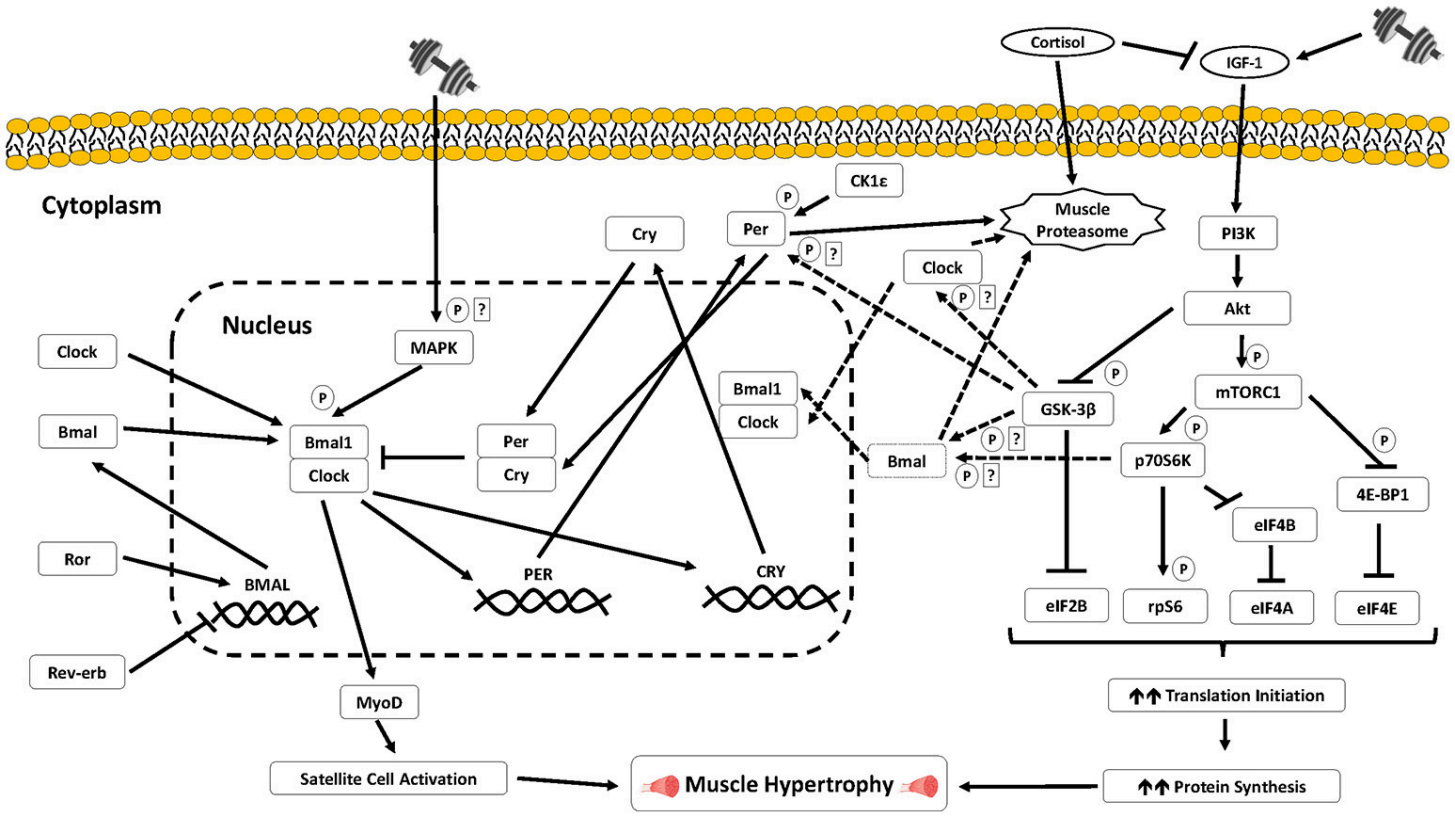
Signaling schematic of the putative pathways that may link the core circadian clock with key signaling mechanisms mediating muscle growth processes in skeletal muscle. Further studies in human skeletal muscle are warranted to ascertain: (a) the potential regulation of the core clock/circadian genes by exercise sensitive signaling proteins and transcription factors in skeletal muscle cells; (b) the potential for the circadian clock to regulate myogenic and translational processes previously associated with heterogeneous anabolic responses following resistance training. *P* indicates phosphorylation; ? indicates potential signaling regulation that requires further experimental validation in skeletal muscle cells.

The molecular clock principally functions to optimize the timing of specific cellular events in anticipation of environmental changes such as daylight and food availability. This occurs through a feedback loop of “core clock genes” involving a series of steps including transcription, translation, post-translational modifications, timed protein turnover, as well as cellular translocation events, that influence the timing of the molecular clock and cellular circadian rhythms. The positive arm of this core loop is operated by the transcription factors CLOCK (circadian locomotor output control kaput) and BMAL1 (brain muscle arnt-like 1) (Hogenesch et al., 1998; Bunger et al., 2000) (Figure 1). These transcription factors heterodimerize and activate transcription of the clock genes Period (Per 1, Per 2, and Per 3) and Cryptochrome (Cry 1 and Cry 2) (Gekakis et al., 1998). CRY and PER subsequently form a complex which forms the negative loop to inhibit the transcriptional activity of the CLOCK: BMAL1 heterodimer. Following transcription and translation, PER1/2 proteins localize to the cytoplasm and can translocate to the nucleus and inhibit CLOCK: BMAL1 transcriptional activity. The orphan nuclear receptors RORα (RAR-related orphan receptor α) and Rev-erb α/β (also known as NR1D1/2; nuclear receptor subfamily 1, group D) comprise additional components of the molecular clock. Rev-erb and ROR are also gene targets of the BMAL1 and CLOCK complex (Preitner et al., 2002; Sato et al., 2004) that can repress and activate BMAL1 and CLOCK transcription, respectively (Preitner et al., 2002; Sato et al., 2004).

The molecular clock is further regulated by several post-translational modifications such as phosphorylation, ubiquitination, and acetylation. Several established kinases, which incidentally are implicated in the molecular regulation of exercise adaptation responses, function together to modulate the period of circadian rhythms by controlling the start, duration, and termination of both the activating and repressing phases of the molecular clock mechanism. Casein kinase 1 epsilon (CK1ε) targets both positive and negative loops. In particular, CK1ε phosphorylates PER1 to induce a conformational change that negates nuclear entry of PER1 that leads to its cytoplasmic accumulation and subsequent degradation (Lefta et al., 2011). By restricting PER1 to the cytoplasm, CK1ε delays PER1-mediated repression of the CLOCK: BMAL1-dependent transcription (Lefta et al., 2011). CK1ε also phosphorylates PER2 and PER3 prior to their ubiquitination and degradation (Akashi et al., 2002; Eide et al., 2005; Shirogane et al., 2005).

Phosphorylation of Glycogen Synthase Kinase 3-β (GSK3-β) targets BMAL1 for phosphorylation followed by subsequent ubiquitination and degradation via the proteasomal pathway (Lefta et al., 2011). Similarly, GSK-3β phosphorylates CLOCK in a modification that primes it for degradation. GSK-3β also phosphorylates PER2 which subsequently enhances its nuclear entry, and triggers the start of transcriptional repression (Sahar et al., 2010). Thus, through phosphorylation of positive and negative limb proteins of the circadian feedback loop, GSK-3β is thought to regulate the onset of both transcriptional activation and transcriptional repression and modulate the period length of circadian rhythms (Figure 1). The mitogen-activated protein kinase (MAPK) can also phosphorylate positive and negative proteins of the molecular clock that regulates the termination of transcriptional activation and duration of transcriptional repression. BMAL1 phosphorylation by MAPK in the nucleus stops CLOCK: BMAL1-mediated transcriptional activation (Sanada et al., 2002). In contrast, MAPK phosphorylation of CRY1 and CRY2 enhances their repression of the CLOCK: BMAL1 heterodimer, lengthening the period of circadian rhythms.

Another central regulatory process of the molecular clock is ubiquitination. The ubiquitin proteasome system involves the “tagging” of proteins to be degraded (Jackman and Kandarian, 2004). Previous work has shown ubiquitin ligases such as SCF (Skp1/cullin/F-box protein) complex (Busino et al., 2007), 26S proteasome (Eide et al., 2005), and Arf-bp1 (Yin et al., 2010) target members of the negative limb of the molecular clock and control their protein turnover by promoting their degradation. Finally, acetylation and deacetylation post-translational modifications are also implicated in the circadian molecular clock. Histone acetyltransferases (HAT) such as p300 and Histone Deacetylase (HDAC) such as Sirtuin 1 (Sirt1) are also involved in the regulation of the molecular clock (Lefta et al., 2011).

## Molecular clock regulation of skeletal muscle function

Skeletal muscle cells express molecular clock genes and show a circadian rhythm expression pattern central to skeletal muscle function including muscle growth and metabolism (Harfmann et al., 2015; Chatterjee and Ma, 2016). It was recently reported *in vitro* that synchronized primary human skeletal myotubes exhibit self-sustained circadian rhythms imperative to the regulation of basal myokine secretion including IL-6 and IL-8 (Perrin et al., 2015). The presence of circadian rhythms in skeletal muscle tissue was initially identified from the discovery of diurnal variations in muscle protein synthesis in rats (Reeds et al., 1986) and later confirmed by transcriptome analyses that demonstrated 215 genes in mouse and 107 in rat skeletal muscle exhibited a circadian rhythmic pattern of expression (McCarthy et al., 2007; Miller et al., 2007). More recent work incorporating high resolution circadian time-course microarray from mice gastrocnemius revealed 1,628 genes exhibit circadian patterns in expression (Hodge et al., 2015). Genes involved in muscle contraction/ growth (MyoD1, Myogenin, Calmodulin 2, Myh1) and metabolism (PDK4, PGC-1β, UCP3) have been shown to display this circadian oscillation in addition to core molecular clock genes such as BMAL1, Nuclear Receptor Subfamily 1 Group D Member 1 and 2, and Per2 (McCarthy et al., 2007; Miller et al., 2007). Subsequent work has also demonstrated skeletal muscle genes displaying circadian rhythms exhibit fiber type dependent expression (Dyar et al., 2015).

The importance of circadian rhythms for skeletal muscle mass, strength, and myofiber type is made evident by the skeletal muscle phenotype observed in models utilizing molecular clock gene-deficient or mutant mice. Indeed, a comparison of skeletal muscle circadian gene expression of clock mutant and control mice demonstrated 30% of cyclic genes display rhythmic perturbations (McCarthy et al., 2007). BMAL1 knockout mice and clock mutant mice present disrupted skeletal muscle myofilament architecture (including decreased expression of the myofibrillar proteins Actin, Myosin, and Titin), fiber-type shifts, decreased mitochondria volume, impaired mitochondrial respiration, and decreased muscle strength at the single-fiber level (Andrews et al., 2010; Dyar et al., 2015). Moreover, muscle-specific BMAL1 knockout mice show a decrease in muscle strength (Dyar et al., 2015; Schroder et al., 2015). In line with a key role of clock proteins in skeletal muscle physiology, Rev-erbα deficiency results in lower exercise capacities due to reduced mitochondrial biogenesis (Woldt et al., 2013). A functional biological circadian clock is also essential to the normal secretion of basal myokines *in vitro* such as interleukin-6 (IL-6), vascular endothelial growth factor (VEGF), and monocyte chemoattractant protein-1 (MCP-1) (Perrin et al., 2015) while recent findings from human skeletal muscle show the biological clock regulates tight rhythms in muscle oxidative metabolism central to the prevention of metabolic-related diseases (van Moorsel et al., 2016). Collectively, these findings show that disruptions in the skeletal muscle molecular circadian clock are strongly associated with altered muscle structure, function, and metabolic responses that directly affect overall health.

## Effects of the skeletal muscle circadian clock on resistance exercise adaptations and molecular responses

Accumulating evidence investigating molecular clock mechanisms and peripheral circadian rhythmicity in skeletal muscle tissues suggests that exercise-induced contraction and its timing may regulate gene expression and protein synthesis related to muscle anabolism and metabolism (Zambon et al., 2003; Schroder and Esser, 2013). Exercise represents a non-photic phase-shifting zeitgeber capable of entraining circadian clocks in skeletal muscles (Van Reeth et al., 1994; Miyazaki et al., 2001; Buxton et al., 2003; Barger et al., 2004; Wolff and Esser, 2012). A recent landmark study demonstrated the importance of “circadian phenotype” on optimal exercise performance by showing the highest performances in aerobic exercise capacity are obtained at different times of the day depending on an individual's chronotype based on sleep-onset time, sleep durations, and wake-up times (Facer-Childs and Brandstaetter, 2015). Compared to endurance-based exercise, the question of whether resistance training anabolic adaptation responses may depend on the time of day of training has currently received little scientific attention. Limited studies report that performing resistance training at a specific time of day could modify the typical diurnal pattern of short-term maximal performances. Souissi and co-workers showed that participants who performed lower body resistance training (twice per week for 6 weeks) in the morning hours (07:00–08:00) increased muscle power more in the morning than in the evening (Souissi et al., 2002). In contrast, participants that trained in the evening hours (17:00–18:00) only improved at this specific time of day suggesting that specific resistance training adaptations are greater at the time of day at which training was conducted than at other times. Moreover, the typical diurnal pattern of maximum isometric strength can be blunted after 10 weeks of resistance training in the morning (07:00–09:00) but not the evening (17:00–19:00) (Sedliak et al., 2007, 2009). Collectively, these results indicate the importance of chronobiology in skeletal muscle adaptations and performance with resistance training although further studies examining the potential for circadian alterations to modulate strength and hypertrophy responses are still required.

The molecular bases for the effects of circadian rhythm on resistance exercise adaptation responses are largely unexplored. Modulations in the mRNA expression of the core molecular clock members and associated translocation events have been implicated, along with hormonal responses. Work from neonatal cardiomyocytes shows CLOCK localizes to the Z-disk within the sarcomere under basal (i.e., non-stimulated) conditions, allowing it to sense fluctuations in energy expenditure associated with contractile activity. Following increased calcium-induced contractile activity, CLOCK translocates to the nucleus to regulate gene expression, suggesting myocardium contraction may directly modulate the molecular clock that in turn regulates cardiac muscle contractile activity (Qi and Boateng, 2006). Murphy and colleagues investigated the capacity for regular exercise performance to regulate the circadian 24-h expression of exercise-relevant genes in skeletal muscle of Thoroughbred mares (Murphy et al., 2014). Mid-gluteal, percutaneous muscle biopsies were obtained every 4 h for 24 h before and after an 8-week exercise program that consisted of 1 h of exercise beginning at 10:30 for 6 days a week. Daytime scheduled exercise was shown to alter the temporal expression pattern of previously identified circadian genes UCP3, MYOD1, and PDK4, indicating that exercise can regulate the entrained patterns of horse skeletal muscle function which may possess direct implications for athletic performance when exercise times are scheduled to coincide with competition times.

To date, only one study has investigated the extent to which resistance exercise can directly modulate circadian-regulated genes in human skeletal muscle as well as the capacity for resistance exercise to regulate diurnally regulated genes. In this study by Zambon and colleagues, DNA microarrays were used to analyse skeletal muscle biopsies from the *vastus lateralis* from 4 middle-aged but healthy males (Zambon et al., 2003). Following a 8-day dietary and physical activity control period, participants commenced a bout of unilateral (i.e., one legged exercise) resistance exercise at 13:30 consisting of 10 sets of eight repetitions of isotonic knee extension at 80% of their predetermined one-repetition maximum. Muscle biopsies were obtained 6 h (between 19:30 and 20:00) and 18 h (between 07:30 and 08:00 the next day) after resistance exercise in both the exercised and non-exercised leg. Results from the gene expression profiles showed that 704 genes and 1,479 genes were differentially regulated at 6 and 18 h after the resistance exercise bout, respectively. Moreover, circadian rhythm displayed the highest Z score from the microarray output with 40% of circadian-rhythm genes classified by the GO hierarchy significantly changed at 6 h after resistance exercise. Three of the core circadian clock genes (CRY1, PER2, and BMAL1) as well as the transcription factor myogenin, were also upregulated 6 h after resistance exercise in the exercised leg only along with select genes involved in nucleic acid metabolism and transcription (Zambon et al., 2003).

These findings present several important advancements including the novel discovery that a single bout of resistance exercise directly transcriptionally regulates core clock gene and circadian output genes. Additionally, this work also showed that peripheral clocks (skeletal muscle in this case) can be regulated independently of the SCN as evidenced by differences in gene expression between the exercised and non-exercised legs. However, there are a number of limitations associated with these findings that must be considered. Firstly, there were only four male participants in this study from a non-homogenous age bracket (31–51 years) that were untrained. Whether the effects of the circadian clock exert similar effects in younger (20–30 years), older (>65 years), highly trained resistance individuals or females is unknown. Another considerable limitation was that there were only two muscle biopsy time points which reduces the capacity to detect peak and trough times in the expression of the circadian-regulated core clock genes. Indeed, the authors failed to detect Cry1 and BMAL1 expression from the two samples obtained. Regardless, available evidence collectively indicates that the regulation of circadian rhythms in skeletal muscle by resistance exercise modulates the molecular profile of adaptation responses that may, over time, influence key physiological responses such as muscle hypertrophy and increased strength.

## Possible implications for the circadian rhythm in heterogeneity of skeletal muscle anabolic responses with resistance training

Considering the current evidence demonstrating the interplay of the molecular clock machinery with growth and hypertrophy responses in skeletal muscle, it stands to reason the circadian clock may also play a critical role in regulating the established molecular mechanisms mediating muscle growth responses with resistance exercise. The premise of a circadian influence on muscle growth is largely based on accumulating evidence showing BMAL1, CLOCK, Rev-erb, and RORα to transcriptionally regulate other genes not directly implicated in circadian function. The downstream targets of these circadian clock genes, termed clock-controlled genes (CCGs) (Bozek et al., 2009; Bass and Takahashi, 2010), are largely tissue-specific (Zhang et al., 2014). Additionally, several tissue-specific transcription factors have been identified as targets of the molecular clock (Andrews et al., 2010; Zhang et al., 2015). Significantly, some of these identified gene targets and transcription factors are prominently involved in the molecular basis associated with myogenesis and muscle protein synthesis responses that have been implicated in anabolic heterogeneity responses with resistance training. Select hormones that can regulate particular molecular pathways implicated in muscle growth and breakdown pathways also exhibit diurnal circadian oscillations and may therefore modulate, in part, anabolic responses with resistance exercise.

### Circadian regulation of myogenic regulatory factors

Andrews and colleagues identified the transcription factor MyoD (myogenic determination factor 1) as a skeletal muscle-specific clock controlled gene through its activation by CLOCK: BMAL1 (Andrews et al., 2010) (Figure 1). MyoD is an established master regulator of the muscle-specific transcription program by promoting withdrawal of myoblasts from cell cycle and the induction of myoblast differentiation (Snijders et al., 2015). The finding by Andrews et al. supports the previous discovery that MyoD mRNA expression oscillates with a circadian profile in rodent skeletal muscle (McCarthy et al., 2007). This oscillation is non-existent, along with the downregulation of specific MyoD controlled genes, in skeletal muscle from both BMAL1^−/−^ and CLOCK^Δ19^ mice (Andrews et al., 2010) as well as in skeletal muscle specific Bmal1 knock out models (Dyar et al., 2014; Hodge et al., 2015). Moreover, BMAL1 deficiency in myoblast cells suppresses myogenesis related-gene expression, including MyoD, Myf5 (myogenic factor 5), and Myogenin expression, and impairs the differentiation of myoblasts to myofibers (Chatterjee et al., 2013). Myogenin, a transcription factor that induces myogenesis by driving the differentiation of somatic cells to fully committed myoblasts that form multinucleated myotubes, also exhibits a circadian pattern of expression similar to MyoD (Shavlakadze et al., 2013).

**Figure 2:**
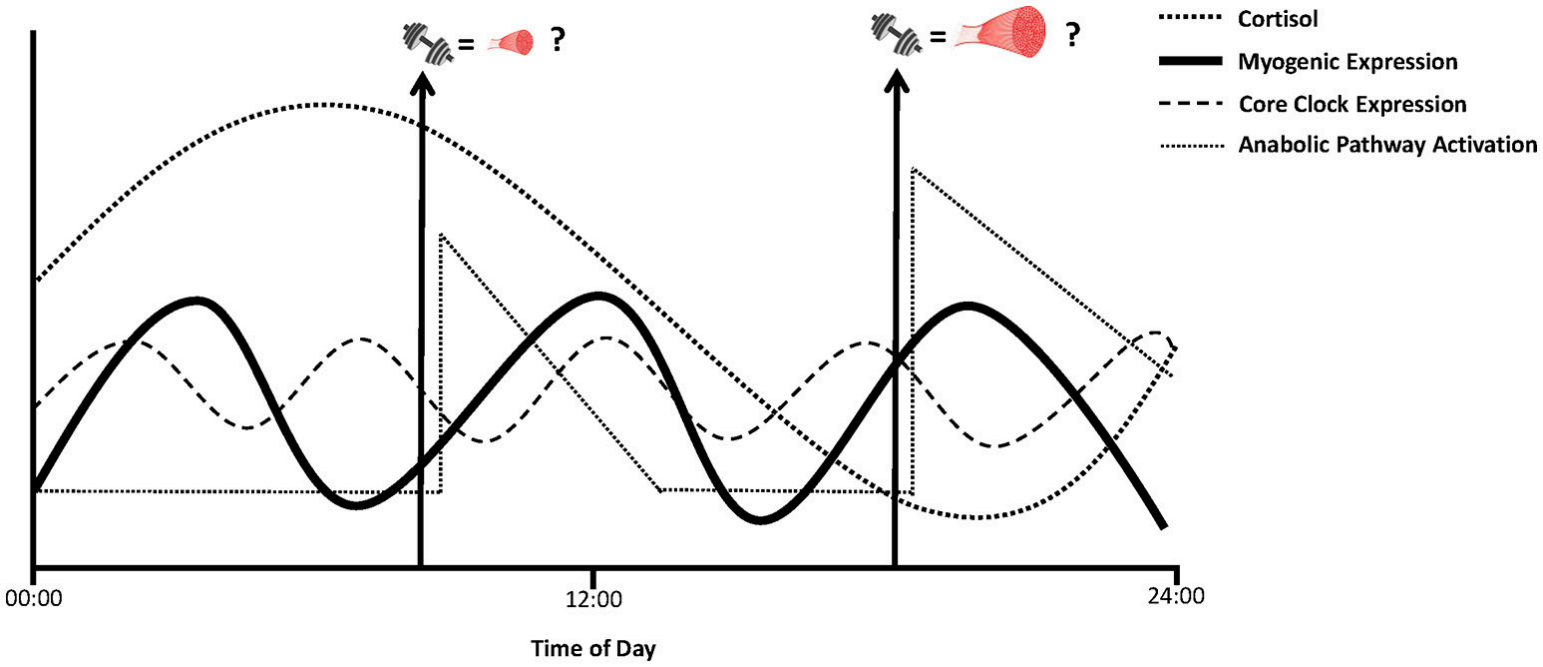
Hypothetical visual of “anabolic periodicity” where the time of day of resistance exercise performance, in conjunction with optimal myocellular (myogenic and translational signaling) and systemic environments, lead to greater anabolic adaptation responses that may, in part, contribute to variable muscle growth, and strength adaptations between different individuals.

As discussed earlier, “extreme” high myofiber hypertrophy responders to a 16-week resistance training program were shown to exhibit either higher basal (i.e., pre-training) or post-intervention increases in MyoD and myogenin transcripts, respectively (Bamman et al., 2007; Thalacker-Mercer et al., 2013). As such, these findings suggest the potential for transient, but chronic, elevations in MyoD and myogenin gene expression with each exercise session are necessary signals that lead to greater muscle hypertrophy induced by resistance training (Bamman et al., 2007). It also raises the possibility that by scheduling bouts of resistance exercise in synchronization with peak diurnal oscillations in expression of these myogenic regulatory factors to promote their maximal activation may, over time, stimulate greater hypertrophy responses (Figure 2). Such a hypothesis requires further extensive investigation incorporating serial muscle biopsy samples and longer term training programs that compare diverse times of day of resistance exercise performance with changes in muscle hypertrophy responses. Nonetheless, this information would provide novel insight to whether the circadian effects on the expression of these myogenic regulatory factors can, for instance, partly explain why “low” or “non-responders” were unable to activate these transcription factors to a level required to induce muscle greater hypertrophy responses.

### Circadian regulation of translational responses

Another prominent and potential signaling mechanism that may link anabolic adaptations following resistance training with the molecular clock is the phosphorylation of BMAL1 by p70S6K1 (ribosomal S6 protein kinase 1). Lipton and colleagues recently showed in mouse embryonic fibroblasts that the phosphorylation of BMAL1 by p70S6K at the Serine 42 site induces BMAL1 to interact with the translational machinery in the cytosol and subsequently stimulate cellular translation independent of BMAL1's established role as a transcription factor (Lipton et al., 2015). The authors also observed that this phosphorylation modification is required for BMAL1 to both interact with cell signaling proteins mediating translation and stimulate protein synthesis, therefore providing a direct molecular link between BMAL1 and the circadian clock to the mTOR signaling cascade translation (Shahbazian et al., 2006).

Phosphorylation of p70S6K in skeletal muscle enhances translation of mRNAs encoding ribosomal proteins and elongation factors and can therefore induce muscle hypertrophy via increases in both translational efficiency and capacity (Wang and Proud, 2006). We (Camera et al., 2010, 2012) and others (Coffey et al., 2006; Dreyer et al., 2006) have previously reported increases in p70S6K phosphorylation in the acute recovery period following resistance exercise. Such increases have also been shown to be predictive of the magnitude of muscle hypertrophy with chronic (i.e., 14–16 weeks) resistance training (Terzis et al., 2008; Mayhew et al., 2011; Mitchell et al., 2013). Thus, the muscle hypertrophy response with resistance training can be partly associated with processes such as the extent of p70S6K phosphorylation following a bout of resistance exercise. Therefore, there is potential that oscillations in BMAL1 may be an important factor for maximizing resistance training-induced muscle growth responses though its association with p70S6K (Figure 1). In particular, the co-ordination of peak resistance exercise-induced increases in p70S6K phosphorylation with elevated diurnal BMAL1 activity may work synergistically to further increase the activation of translational machinery necessary to stimulate elevated rates of muscle protein synthesis which form the basis for greater increases in muscle growth overtime (Figure 2).

### Circadian regulation resistance exercise hormone responses

Select hormones can regulate the activation of signaling pathways that regulate muscle cell metabolism, structure, and function. Several hormones, in particular cortisol, and testosterone, reveal different diurnal oscillations with purported implications in anabolic adaptation responses with resistance training (Hayes et al., 2010; Chtourou and Souissi, 2012). Considering current evidence strongly indicating transient increases in “anabolic hormones,” namely testosterone, do not enhance muscle protein synthesis responses following resistance exercise (West et al., 2009, 2010), this section will focus on the capacity for circadian oscillations in cortisol to modulate adaptation processes with resistance exercise. Under resting/not stimulated conditions, cortisol peaks upon morning wakening, followed by a decline across the day, reaching its lowest level ~3–5 h after night-time sleep onset (Hayes et al., 2010). Cortisol is associated with muscle catabolism through the activation of the ubiquitin-proteasome pathway (Auclair et al., 1997; Jackman and Kandarian, 2004). Cortisol also inhibits the production of the hormone insulin growth factor 1 (IGF-1) (Dattilo et al., 2011). Increases in IGF-1 in response to skeletal muscle contraction activate the type I IGF receptor (IGF-IR) (Clemmons, 2009) which can subsequently activate downstream signaling targets, including mTORC1 and p70S6K1 through the insulin/IGF-1-phosphatidylinositol 3-kinase (PI3K)-Akt signaling pathway, implicated in exercise-induced increases in skeletal muscle hypertrophy (Bodine et al., 2001; Clemmons, 2009). Moreover, animal studies have shown IGF-I can play a role in the differentiation and fusion of satellite cells (Benito et al., 1996).

The established circadian flux of cortisol raises the prospect that the specific timing at which resistance exercise is performed to coincide with nadirs in cortisol secretion can play a role in the magnitude of anabolic response obtained. For instance, by performing resistance exercise at a time when cortisol levels are low may allow for increased circulatory secretion of IGF-1 to therefore allow the subsequent activation of p70S6K central to the promotion of translation initiation responses. Several studies have indicated that the performance of resistance exercise in the evening compared to the morning is associated with lower circulating cortisol levels (Ahtiainen et al., 2003; Bird and Tarpenning, 2004; Burley et al., 2016), indicating the potential for evening over morning for reduced catabolic environments to promote muscle hypertrophy adaptations. Burley et al. investigated the effect of a single bout of resistance training performed either in the morning (08:00) or evening (18:00) on circulating cortisol levels and *in vitro* myogenic differentiation (Burley et al., 2016). In agreement with previous work, they found cortisol levels were significantly lower in the evening compared to morning conditions pre-and post-exercise. Notably, Burley et al also observed an elevated myogenic index and myotube width in the mouse C2C12 cell line treated with serum from the same participants obtained from the PM exercise session compared to AM exercise condition which the authors postulate may be a result of the lower evening cortisol levels. These findings collectively indicate the potential for a cellular environment more conducive to enhancing muscle anabolism when resistance exercise is performed in the evening compared to morning (Figure 2). In regards to muscle-centric mechanisms, it was recently reported after an 11-week training period in untrained individuals that p70S6K phosphorylation on the Thr421/Ser 424 residue significantly increased following morning, but not afternoon, resistance exercise (Sedliak et al., 2017). This divergent time-of-day phosphorylation response occurred despite a trend for higher cortisol levels in the morning compared to afternoon. Nonetheless, both morning and afternoon training groups presented similar increases in strength, thigh cross-sectional area and fiber cross-sectional area (Sedliak et al., 2017). Moreover, there were no differences in other anabolic signaling markers measured, including phosphorylation of p70S6K at the Thr389 site which has been shown to most closely correlate with p70S6 kinase activity *in vivo* (Weng et al., 1998). Direct measures of rates of muscle protein synthesis will provide greater insight to the extent, if any, that cortisol may impact anabolic adaptations with resistance exercise undertaken in the morning compared to evening (and vice-versa). Moreover, resistance training performed consecutively in the morning can actually decrease circulating cortisol levels over time (Sedliak et al., 2007). This response, which may indicate a gradual habituation to the “stress” imposed exercise in the morning, suggests the systemic environment to promote optimal anabolic adaptations with resistance training may be modifiable by the consistent, repeated performance of resistance training in the morning (Sedliak et al., 2007). This may be of benefit to individuals who can only perform resistance training in the morning due to lifestyle limitations (work, family, etc.).

In summary, evidence exists that links key muscle signaling and systemic/circulatory events possessing circadian rhythms with the circadian molecular clock that may impact the degree of skeletal muscle adaptation responses with resistance training. While these associations require much further investigation incorporating human skeletal muscle and resistance protocols with repeated muscle biopsies that control for circadian zeitgebers and divergent chronotypes, it must be noted that there are several limitations with the hypothetical associations discussed. For instance, the necessity for activation of MyoD to regulate the magnitude of muscle fiber cross sectional response with resistance exercise is questionable. As evidenced in the work by Bamman et al. MyoD transcript levels presented similar increases between “extreme,” “modest,” and “non-responder” cohorts post-resistance training (Bamman et al., 2007). The correlation between resistance exercise induced-increases in p70S6K phosphorylation and changes in muscle growth is also equivocal. Indeed, this association is strictly time-course dependent (Mitchell et al., 2013) or not even present all (Mitchell et al., 2012). Finally, with regards to the potential for cortisol to regulate muscle growth responses with resistance training, considerable evidence questions the role of transient fluctuations in endocrine factors to modulate anabolic signaling or muscle protein synthesis responses with several lines of evidence indicating local (intra-muscular) mechanisms to be the predominant contributor to muscle growth responses (West et al., 2009, 2010). However, perhaps the greatest overall limitation is that these studies that present findings that may contradict the potential discussed associations between the molecular mechanisms mediating exercise adaptation responses and circadian rhythms have not strictly controlled for circadian oscillations in their experimental design. As such, it is unknown how differences in individual participant chronotype or if a consistent time of day for exercise and muscle biopsy sampling were included may influence the results obtained from this previously published work.

## Conclusion and future directions

Circadian studies in skeletal muscle are critical for understanding the effects of circadian clock gene regulation on the molecular mechanisms mediating adaptation responses with exercise. However, before the molecular mechanisms underlying the role of the circadian clock in exercise adaptations responses can be accurately defined, further work in rodents where factors such as light exposure, feeding, temperature, sleep quantity and quality, and genetic predisposition can be better controlled (compared to the human model) are firstly required. Additionally, most studies investigating the capacity for exercise to alter circadian rhythms have centered on endurance exercise and much less is known about the potential for resistance exercise to modulate molecular clock function in skeletal muscle. The optimal time of day to perform resistance exercise to induce the greatest synergistic anabolic effect of resistance exercise on myogenic regulatory factor expression, translational signaling transduction and optimal hormonal milieu responses is unknown. This information could provide novel insight to the mechanisms that may contribute to heterogeneity in anabolic-related adaptation responses (i.e., myofiber cross sectional area, strength, etc.) with resistance training as well as progress the future development of individualized exercise training programs to maximize adaptation responses. Additionally, the potential for an “optimal” time to perform resistance exercise in conjunction with circadian clock pathways and hormonal regulation raises the prospect of an “anabolic periodicity” such that certain times of the day may provide a greater overall cellular and systemic environment to maximize resistance exercise adaptation responses. This proposal is of practical relevance to strength and powerlifting athletes who are needed to maximize anabolic training outputs for optimal performance outcomes.

**Figure 3:**
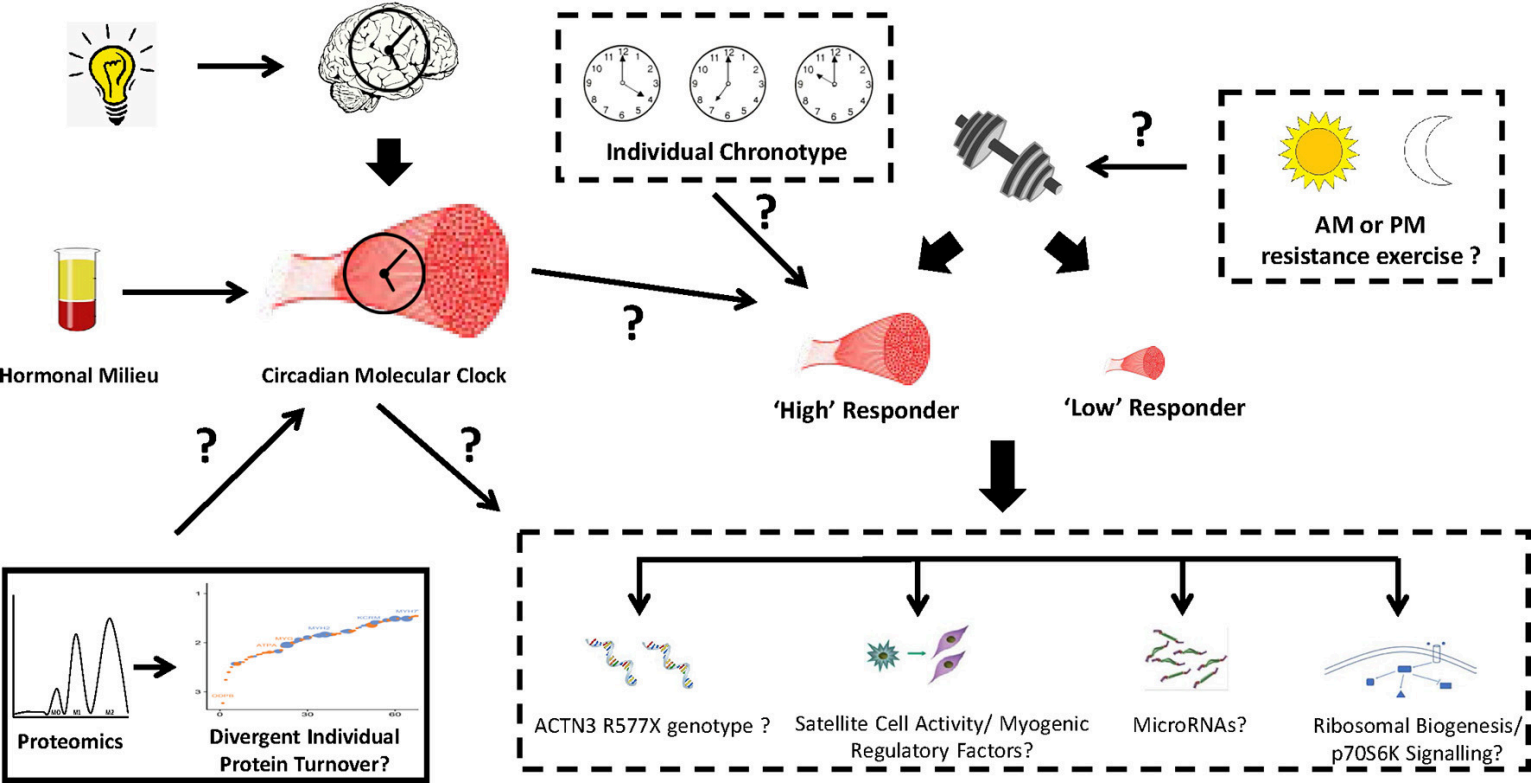
Hypothetical link between the skeletal muscle molecular clock and resistance exercise adaptation responses. Several molecular mechanisms have been implicated in the molecular basis associated with anabolic heterogeneity following resistance exercise. Scheduled exercise can entrain the circadian clocks in skeletal muscle, however it is unknown whether the time of day performance of resistance exercise (morning, afternoon, or evening), an individual's sleep/activity chronotype, and the associated effects on the molecular clock, can influence anabolic adaptations with resistance training. An area of further investigation within this paradigm is the use of proteomic platforms to interrogate whether the turnover rates of myofibrillar and sarcoplasmic proteins are differentially altered when resistance exercise is performed in the morning or evening. This information would provide valuable insight to the molecular mechanisms that govern anabolic adaptations with resistance training and help decipher whether particular individuals may experience greater benefits with resistance training when performed at different times of the day.

Several areas of further investigation remain in this emerging field of circadian clock and regulation of the mechanisms mediating the degree of anabolic responses with resistance training. First and foremost, the incorporation of study designs that strictly control the time of day when both muscle biopsy samples are collected and participants perform exercise within controlled environmental settings are warranted to limit inherent variability in circadian rhythms between individuals. In extension of this, the development of new methods that objectively assess individual chronotype as a means to more accurately assess the importance of the molecular clock on anabolic responses to resistance exercise is needed. Secondly, work in mice has shown circadian genes to be differentially expressed between fast (Type II) and slow (Type I) fibers which is likely a result of established differences in activity levels, substrate metabolism and functional properties between muscle fiber types (Dyar et al., 2015). Whether the same disparities exist among different muscle groups in humans is unknown. In this regard, studies that have investigated muscle-centric mechanisms implicated in the divergent magnitude of anabolic response with resistance training have only focused on the *vastus lateralis*. Thus, additional analyses in other activated muscle groups that involves the simultaneous investigation of the circadian molecular clock may yield new insight to additional mechanisms underlying heterogeneity in muscle growth responses with resistance training. Another relevant factor in the circadian regulation of anabolic responses with resistance exercise is the interaction with nutrients. Feeding of nutrients, like exercise, is a known entrainment factor of skeletal muscle (Wolff and Esser, 2012; Bray et al., 2013). The consumption of liquid and whole-food protein sources is well established to enhance muscle protein synthesis responses following resistance exercise (Phillips et al., 2005). Optimizing the timing of combined resistance exercise performance and defined protein-ingestion patterns could have the potential to modulate the circadian clock in a way that provides an ideal muscle and systemic environment conducive to maximal muscle growth responses. Finally, the use of innovative technology platforms such as RNA sequencing and proteomics to investigate the molecular mechanisms implicated in muscle growth responses with resistance training may also lead to novel information related to skeletal muscle molecular clock function with resistance exercise (Figure 3). Integrating proteomic profiling with deuterium labeling, we recently showed that rates of muscle protein synthesis and breakdown following short-term resistance training differ on a protein-by-protein basis (Camera et al., 2017). Whether protein-specific synthesis and breakdown responses are altered by the circadian clock when resistance exercise is performed at different times of the day is unknown. Enhanced or dysregulated protein turnover responses in this context may be implicated in the known variability in myofiber hypertrophy responses with resistance training (Figure 3).

## Author contributions

The author confirms being the sole contributor of this work and approved it for publication.

### Conflict of interest statement

The author declares that the research was conducted in the absence of any commercial or financial relationships that could be construed as a potential conflict of interest.
